# The effect of protein and essential amino acid supplementation on muscle strength and performance in patients with chronic heart failure: a systematic review

**DOI:** 10.1007/s00394-019-02108-z

**Published:** 2019-10-28

**Authors:** Simon Nichols, Gordon McGregor, Abdallah Al-Mohammad, Ali N. Ali, Garry Tew, Alasdair F. O’Doherty

**Affiliations:** 1 Centre for Sports and Exercise Science, Sheffield Hallam University, Collegiate Campus, Sheffield, S10 2BP UK; 2 Centre for Exercise and Health, Department of Cardiopulmonary Rehabilitation, University Hospitals Coventry and Warwickshire NHS Trust, Coventry, UK; 3 School of Health and Life Sciences, Coventry University, Coventry, UK; 4 Sheffield Teaching Hospitals NHS Foundation Trust, Herries Rd, Sheffield, UK; 5 Sheffield National Institute for Health Research Biomedical Research Centre, Glossop Road, Sheffield, UK; 6 Department of Sport, Exercise and Rehabilitation, Northumbria University, Newcastle-Upon-Tyne, UK

**Keywords:** Heart failure, Sarcopenia, Cachexia, Frailty, Muscles, Amino acids, Diet

## Abstract

**Purpose:**

Critically low skeletal muscle mass and strength, observed in 20% of people with chronic heart failure (CHF), reduces functional capacity, quality of life (QoL) and survival. Protein and essential amino acid (EAA) supplementation could be a viable treatment strategy to prevent declines in muscle strength and performance, and subsequently improve QoL and survival. This systematic review (PROSPERO: CRD42018103649) aimed to assess the effect of dietary protein and/or EAA supplementation on muscle strength and performance in people with CHF.

**Methods:**

Searches of PubMed, MEDLINE and Embase identified studies that reported changes in strength or muscle performance following protein and/or EAA supplementation in patients with CHF. Following PRISMA guidelines and using predefined inclusion/exclusion criteria relating to participants, intervention, control, outcome and study design, two reviewers independently screened titles, abstracts and full manuscripts for eligibility. Risk of bias was assessed using Cochrane Risk of Bias Tool (RCTs) or Mixed Methods Appraisal Tool (cohort studies). Data were extracted for analysis using predefined criteria.

**Results:**

Five randomised controlled trials (RCT) and one cohort study met our inclusion criteria. All RCTs had a high risk of bias. The methodological quality of the cohort study was moderate. Heterogeneity of extracted data prevented meta-analyses, qualitative synthesis was therefore performed. Data from 167 patients with CHF suggest that protein and/or EAA supplementation does not improve strength, but may increase six-minute walk test distance, muscle mass and QoL.

**Conclusions:**

The limited quality of the studies makes firm conclusions difficult, however protein and/or EAA supplementation may improve important outcome measures related to sarcopenia. High-quality randomised controlled studies are needed.

**Electronic supplementary material:**

The online version of this article (10.1007/s00394-019-02108-z) contains supplementary material, which is available to authorized users.

## Introduction

Chronic heart failure (CHF) affects 11.8% of people over the age of 60 years [1] and is a leading cause of death and disability [2]. Structural and functional cardiac abnormalities, leading to imbalances between metabolic supply and demand, are the defining physiological characteristics of CHF [3]. A key phenotype of CHF is reduced cardiac output (*Q̇*) and arterial compliance, which collectively inhibit haemodynamic perfusion of skeletal muscle during physical activity [3]. Such impaired cardiovascular function may contribute to exercise intolerance; however, CHF also causes profound adverse changes to skeletal muscle physiology, which plays a significant role in mediating physical disability [3].

Changes in skeletal muscle physiology occur in CHF patients who have either reduced (HFrEF) or preserved (HFpEF) left ventricular ejection fraction (LVEF) [4–8]. These changes have been described in detail elsewhere [4, 9] and include a decrease in the number of type I muscle fibres, a decrease in the oxidative capacity and cross-sectional area of type II muscle fibres, a reduction in mitochondrial volume within muscle fibres, a reduction in enzymes required for aerobic metabolism and an increase in glycolytic enzymes. Reduced skeletal muscle aerobic enzyme activity, mitochondrial density and perfusion matching with oxidative muscle fibres contribute to poor aerobic fitness, faster depletion of phosphocreatine and an earlier reliance on glycolytic pathways during exercise [4]. Patients with CHF are also more likely to suffer from muscle atrophy and reduced strength, a condition termed sarcopenia [10–12]. In CHF, sarcopenia is associated with an increased risk of premature mortality [13], a reduction in six-minute walk test (6MWT) distance and physical function, low aerobic fitness, and poor health-related quality of life (HRQoL) [11, 14]. Treatments that are capable of reversing or preventing the development of sarcopenia in patients who have CHF are therefore needed.

Insufficient dietary protein intake is a strong predictor of developing sarcopenia in patients with heart disease [15]. Regular dietary supplementation with protein or essential amino acids (EAA) has been shown to augment skeletal muscle strength and mass in healthy adults [16], and in patients with a long-term condition; defined by the authors as, “including coronary artery disease, chronic heart failure, type 2 diabetes mellitus, chronic obstructive pulmonary disease, osteoporosis, the metabolic syndrome and dementia” [17]. This is most likely due to the capacity of dietary protein and/or EAA supplementation to stimulate the mammalian target of rapamycin (mTOR) pathway and muscle anabolism [18–20]. Protein and/or EAA supplementation may therefore help to improve strength and muscle performance in patients with CHF, however this has not been widely investigated.

The primary aim of this systematic review was to assess the effects of dietary protein and/or EAA supplementation on skeletal muscle strength and performance in people with CHF. The secondary aims were to explore the effect of this intervention on body composition, HRQoL, aerobic fitness and safety. Intervention adherence and adverse events were also reported.

## Methods

This review adhered to the Preferred Reporting Items for Systematic Reviews and Meta-Analyses (PRISMA) guidelines. A PRISMA checklist is available from Online Resource 1 [21]. A priori aims, eligibility criteria and methods were registered with PROSPERO (CRD42018103649).

### Study selection criteria

The Participants, Intervention, Control, Outcomes and Study Type (PICOS) criteria are outlined in Table 1. Randomised controlled trials (RCT) and cohort studies were included if the intervention involved protein or EAA supplementation for at least 4 weeks. Protein supplementation was defined as a request for the participant to consume protein and/or EAA in addition to their habitual dietary intake. We conservatively chose to include studies that had a supplementation period of at least 4 weeks because a recent systematic review that investigated protein supplementation in patients with a long-term condition, suggested that interventions as short as 6 weeks may improve muscle strength and mass [17]. Further inclusion criteria were: studies that recruited (1) male or female patients, (2) patients who were > 18 years, and (3) patients with a diagnosis of HFrEF or HFpEF. Studies were also required to have a primary outcome of skeletal muscle strength or performance. Examples of performance outcome measures included walking tests, the short physical performance battery (SPPB) or gait speed, because these have been recommended in international sarcopenia guidelines [10]. Acceptable comparator groups included patients that were assigned to standard care (no change to diet) or the modification of a patient’s diet that resulted in a lower protein intake in comparison with the intervention group.

**Table 1:** PICOS criteria for included studies

PICOS	Criteria
Participants	Heart failure patients with preserved ejection fraction
	Heart failure patients with reduced ejection fraction
Intervention	Dietary protein supplementation for at least 4 weeks
	Dietary essential amino acid supplementation for at least 4 weeks
Control	Standard medical care (no change in diet)
	Modification of a control patient’s diet resulting in a lower protein intake, compared to intervention patients
Outcomes	Muscle strength
	Muscle performance
Study type	Randomised controlled trial
	Cohort study

Studies were excluded if: (1) research was conducted in animal models, (2) participants were < 18 years of age, (3) there was no dietary protein or EAA supplementation, (4) the supplementation period was less than 4 weeks or (5) data were from case reports. Systematic reviews were also excluded. Language of publication was not an exclusion criterion.

### Search strategy

A search of PubMed, MEDLINE, and Embase, was conducted from the dates of inception (1879, 1879 and 1947, respectively), to August 2018. Search terms, including medical subject headings (MeSH) were developed by SN and AOD. These were refined by an independent research librarian who performed the literature search. The search strategy combined keywords describing the primary condition [heart failure (MeSH) OR cardiac failure OR left ventricular failure] AND secondary condition [sarcopenia (MeSH) OR cachexia (MeSH) OR lean muscle OR muscle mass]. The strategy also included terms describing the ‘intervention’ [protein and amino acids (MeSH)]. There are numerous methods of assessing strength and muscle performance. To reduce the risk of excluding relevant articles, we did not restrict the outcome measures using specific search terms. A full search strategy is provided in Online Resource 2. The reference lists of manuscripts that met our inclusion criteria were also screened for articles that met our inclusion criteria (see below).

### Study selection

After the search was completed, duplicate articles were removed. SN and AOD independently screened titles and abstracts in accordance with inclusion and exclusion criteria. Full-text manuscripts of the abstracts that met our inclusion criteria were assessed against inclusion and exclusion criteria. Differences between the author’s lists of included studies were resolved through discussion between SN, AOD and ANA, at both stages of the review process. Data extraction was performed by SN using a pre-established pro forma. AOD reviewed the extracted data against the original manuscripts.

### Data extraction and analysis

The following data were extracted: muscle strength, muscle performance, body composition, aerobic fitness, HRQoL, age, sex and number of participants, LVEF, primary diagnosis (HFrEF or HFpEF), and description of the supplementation regime (type of supplement, dose, frequency and duration), attrition, adherence, and safety (adverse events, renal function). Transparent reporting of adverse events provides important context about the benefit and risk profile of an intervention and should be an outcome reported in clinical trials [22]. Serious adverse events were defined as any event or reaction that resulted in death, life-threatening illness, hospital admission or prolongation of existing hospitalisation, persistent or significant disability or incapacity [23]. Adverse events were defined as an untoward medical event that occurred during activities required for the study [23], irrespective of whether they were thought to be related to the intervention. Changes in renal function were also reported to explore the safety of protein and/or EAA supplementation in patients with CHF. Diets that are high in protein may be associated with a decline in renal function in patients with heart disease [24]. Where outcome measures were assessed at multiple time points, testing conducted closest to cessation of supplementation was included for analysis. Attempts were made to contact corresponding authors when missing data were identified.

We planned to conduct meta-analysis on quantitative data extracted from included studies. Outcomes of interest were extracted as inter-group mean difference, with standard deviation (±), or median with inter-quartile range (IQR), according to how they were reported in the original manuscript. Intra-group differences were reported, where inter-group differences were unavailable. Data dispersion reported as standard error of the mean (SEM) were converted to standard deviations using the following equation:

$${\text{SD}}\,{ = }\,{\text{SEM}}\, \times \,\sqrt {n\,}$$

where; SD is the standard deviation, SEM is the standard error of the mean and *n* is the number of participants in the group of interest. Improvements in an outcome variable were considered statistically significant if it achieved a significance threshold outlined in an a priori sample size calculation. Where sample size calculations were not provided, findings were considered significant if a *P* value < 0.05 was reported.

### Risk of bias and quality appraisal

Randomised controlled trials (RCTs) meeting our study inclusion criteria were independently evaluated by SN and AOD for risk of bias, using the Cochrane Risk of Bias tool [25]. Bias attributed to patient selection, randomisation, blinding, attrition and data reporting was assessed. Studies with a high risk of bias in one or more domains were classified as high risk. Studies that had an unclear risk of bias in one or more domains, but were not considered to have any domains at high risk of bias, were classified as moderate risk. Any study meeting low-risk criteria for all domains was considered to be at low risk of bias. A methodological appraisal of cohort studies was undertaken by SN and ANA using the Mixed Methods Appraisal Tool (MMAT) [26]. Objective quality scoring is not recommended because it assigns unjustified weighting to different elements of trial design and reporting [25]. A subjective assessment was therefore undertaken (Online Resource 3).

## Results

**Fig. 1:**
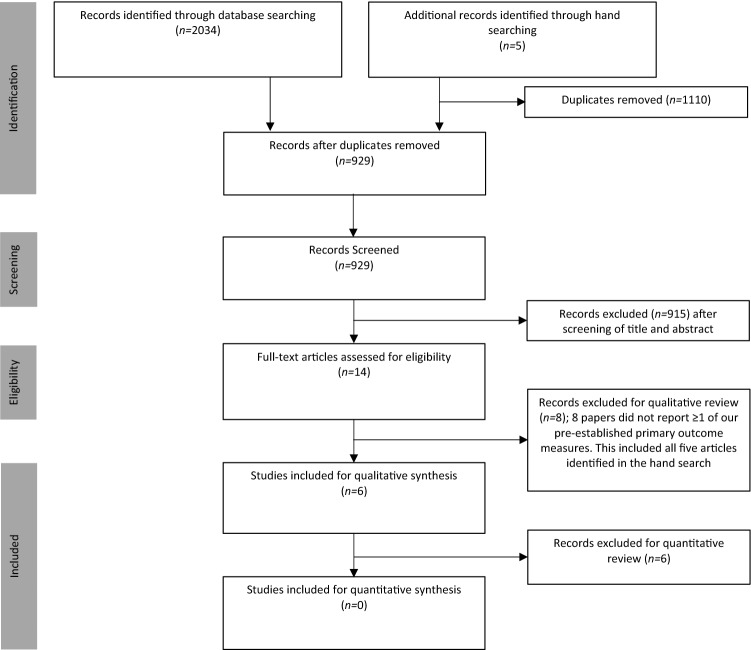
PRISMA flow chart

A PRISMA flow diagram is shown in Fig. 1. Searches identified *n *= 833, *n *= 469 and *n *= 732 articles from Embase, MEDLINE and Pubmed, respectively (total *n *= 2034). After the removal of duplicate articles (*n *= 1110), database searches identified 924 records. A further five articles were identified through hand searches (total *n *= 929). Fourteen full-text articles were retrieved after screening of titles and abstracts. Eight articles, including the five studies identified in the hand search, were excluded (Online Resource 4) and six articles were retained for review [27–32]. Data reported within these manuscripts was heterogeneous and there was insufficient data to perform meta-analysis or quantitative synthesis due to the risk of generating misleading findings [33]. Data were therefore qualitatively synthesised. No authors responded to our request for further information.

### Study characteristics

Study characteristics are reported in Table 2. One cohort study [32] and five RCTs [27–31] were identified. One-hundred and three (*n *= 103) patients were recruited to intervention groups and 64 patients were recruited to control groups, providing a total population of 167 patients from six studies. One RCT did not report primary outcome data for their control group [28].

**Table 2:** Characteristics of included studies (values reported as mean ± standard deviation unless otherwise specified)

Study	Year	Country of publication	Journal	Design	Criteria for CHF	Sample size (*n *=)	Mean age (years)	SD of age (years)	Number of males (%)	Intervention (I) and control (C)	Supplement duration	Primary outcome(s)	Adverse events
Aquilani et al. [27]	2008	Italy	European Journal of Heart Failure	RCT	‘Muscle depleted’ patients with clinically stable CHF	I: 21 C: 17	I: 73 C: 75	I: 5 C: 3	I: 13 (62) C: 14 (82)	I: 2 × 4 g essential amino acid drink. C: Standard medical care.	2 Months	Increase in body mass > 1 kg over 2 months	I: 0 SAE; 1 AE C: 3 SAE; 0 AE
Rozentryt et al. [28]	2010	Poland	Journal of Cachexia Sarcopenia and Muscle	RCT	Cachectic, NYHA II–IV, LVEF ≤ 30%, oedema free weight loss > 7.5% over > 6 months	I: 23 C: 6	I: 52 C: 49	I: 10 C: 12	I: 17 (74) C: 5 (83)	I: 600 kcal (20 g protein, 72 g cho, 26 g fat) divided into two equal doses. C: 12 kcal placebo control meal of similar taste consistency	3 months	Oedema-free body mass and HRQoL	I: 11 SAE; 16 AE C: 3 SAE; 7 AE
Pineda-Juarez et al. [29]	2015	Mexico	Clinical nutrition	RCT	Stable CHF according to ESC guidelines [34]	I: 29 C: 26	I: 75 (median) C: 71 (median)	I: 64–84 (range) C: 58–79 (range)	I: 19 (56) C: 14 (44)	I: 2 × 5 g BCAA servings per day. Dietary protein consumption standardised to 20% of total estimated daily energy intake. Resistance exercise training 2 × 1/h per week. C: Dietary protein consumption standardised to 20% of total estimated daily energy intake. Resistance exercise training 2 × 1/h per week	12 weeks	Not specified	I: 2 SAE; No reported AEs C: 2 SAE; No reported AEs
Wu et al. [30]	2015	USA	Circulation: heart failure	RCT	LVEF ≤ 35%	I: 14 C: 12	I: 59 C: 56	I: 3 C: 2	I: 12 (86) C: 9 (82)	I: 8 g/day L-alanyle-l-glutamine and 6.5 g/day fish oil C: Safflower oil and milk powder of equivalent caloric intake	6 weeks	Change in CPET, 6MWT and isokinetic and isometric muscle function	I: SAEs; 2 AEs C: SAE; 2 AEs
George et al. [31]	2017	USA	Journal of physiotherapy and physical rehabilitation	RCT	NYHA II–III, HFpEF and/or HFrEF	I: 3 C: 3	I: 84 C: 75	I: 1 C: 7	Not reported	I: Whey isolate powder supplement to increase protein intake to 1.5 g/kg body mass per day and exercise DVD including aerobic and resistance exercise 6 days per week. C: Standard medical care	6 months	Not specified	Not reported
Lombardi et al. [32]	2014	Italy	Clinical Medicine Insights: Cardiology	Cohort	NYHA II–III, LVEF < 45%	I: 13	I: 59	I: 14	I: 11 (85)	I: 2 × 4 g sachets contain 11 essential and semi-essential amino acid per day	3 months	CPET and 6MWT distance	Not reported

*I* intervention group, *C* control group, *CHF* chronic heart failure, *SD* standard deviation, *RCT* randomised controlled trial, *NYHA* New York heart failure classification, *HFpEF* heart failure with preserved ejection fraction, *HFrEF* heart failure with reduced ejection fraction, *LVEF* left ventricular ejection fraction, *CPET* cardiopulmonary exercise test, *6MWT* six-minute walk test, *CHO* carbohydrate, *HRQoL* health-related quality of life, *SAE* serious adverse event, *AE* adverse event

Patient inclusion criteria were different between studies. Reduced LVEF or New York Heart Failure (NYHA) classification II–IV was most frequently reported as the criteria to define heart failure [28, 30, 32]. Two studies specifically recruited patients with clinical signs of muscle depletion [27, 28]. Muscle depletion was defined as: > 7.5% oedema-free weight loss in ≥ 6 months, excluding patients with signs of acute inflammatory processes, cancer, or severe chronic renal failure (serum creatinine > 250 μmol/L) [25], or age and sex adjusted arm circumferences in the lowest 10th percentile in accordance with data from Frischano and colleagues [35].

### Risk of bias

All five RCTs had a high risk of bias (Table 3) [27–31]. Randomised controlled trials had between two and six domains that were considered to be at a high risk of bias. The methodological quality of the cohort study was considered moderate due to the length of the recruitment not being reported (Online Resource 3) [32]. Selective data reporting was common across all studies and numerical *P*-values were often unreported. Only one study reported a sample size calculation [30].

**Table 3:**
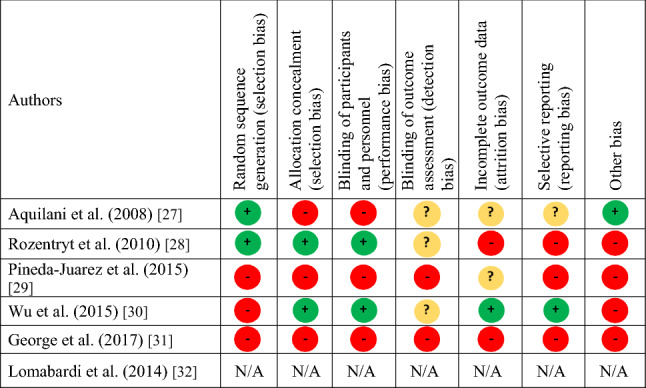
Risk of bias table for included studies

### Duration of protein and essential amino acid supplementation

Intervention characteristics (Table 2) were heterogeneous. The duration of dietary supplementation of protein/EAA ranged from 6 weeks [30] to 6 months [31]. Three studies supplemented protein/EAA for 3 months [28, 29, 32] and one study supplemented protein/EAA for 2 months [27].

### Daily protein and essential amino acid dose

Five out of six studies increased daily dietary protein and/or EAA intake with supplementation. Among these five studies, two administered dietary protein; one used 1.5 g/kg per day of whey protein powder [31] and one used a 300 kcal of twice daily multi-macronutrient supplement containing 20 g of protein [28]. Three of the five studies administered an EAA supplement without further alteration to the patient’s habitual macro, or micro nutrient intake. Two of these provided 4 g doses, twice daily [27, 32] and one administered an 8 g dose, once daily [30].

Instead of increasing daily dietary protein and/or EAA intake by the same (relative or absolute) amount, one of the six studies adjusted daily dietary protein intake to meet 20% of a patient’s daily estimated energy intake in both the intervention and the control groups [29]. Ten grams of protein was then removed from the daily diets of patients in the intervention group and replaced with 2 × 5 g of a combined EAA and non-EAA supplement [29]. Supplement doses are shown in Table 2.

### Combined interventional studies

One study combined protein supplementation with a home-based aerobic and resistance exercise training intervention [31]. Exercise was delivered via a DVD or pamphlet and aimed to improve ambulation, balance, lifting and functional independence. Each 20-min exercise session was prescribed six times per week (three aerobic and three resistance exercise sessions on alternating days). This exercise training intervention was only prescribed to the supplemented group.

A second study combined EAA supplementation with a twice weekly (1 h per session) resistance exercise training programme [29]. Resistance exercise training consisted of a warmup that included mobility exercises (four sets of six different exercises) and skeletal muscle stretching (four sets of six different exercises). The conditioning phase also involved six different exercises. Each type of resistance exercise was conducted in four sets of 15–20 repetitions. Barbell and exercise bands providing a resistance between 500 and 1500 g were used. Both the control and the supplement intervention groups undertook this exercise protocol.

One study combined EAA (8 g/day) with 6.5 g/day of polyunsaturated fatty acid (fish oil) [30].

### Control group characteristics

Control group designs varied between studies. Two control groups were provided with standard medical care (no placebo) [27, 31]. One received safflower oil and milk powder of equivalent caloric intake [30]. Another received the same resistance exercise training programme provided to the intervention group, whilst having their dietary protein intake adjusted to 20% of their estimated daily energy intake [29]. Only one control group was provided with a placebo drink (12 kcal) of ‘similar taste and consistency’ [28].

### Strength measurements

Three studies with a combined total of *n *= 46 intervention patients and *n *= 41 control patients investigated changes in skeletal muscle strength [29–31] (Table 4). One of these studies included exercise prescription in the intervention group only [31], one study included exercise prescription in the intervention and control groups [29] and one study did not prescribe exercise [30]. One study reported percentage changes in handgrip strength only [29], one reported changes in handgrip strength (numerical data unavailable) and isokinetic leg dynamometry [30] and one reported both handgrip (kg) and quadriceps (kg) strength, but did not provide information of how measurements were recorded [31]. No studies reported any statistically significant improvements in strength in patients assigned to the intervention, compared to patients in control groups.

**Table 4:** Changes in strength measurements (mean ± SD)

Study	Sample size (*n *=)	Measurement of strength	Mean change in strength (SD)	Significant improvement
Pineda-Juarez et al. [29]	I: 29 C: 26	Handgrip (dominant arm; kg)	I: + 8.0 (− 6.8 to 15.3)^a^ C: + 11.4 (3.6 to 21.4)^a^	I: No—*P *> 0.05 C: No—*P *> 0.05 Inter-group: Not reported
Wu et al. [30]	I: 14 C: 12	Isometric leg extension—peak torque/BW (%)	I: 196.0 ± 37.4 to 206.0 ± 41.2 C: 177.0 ± 62.4 to 200.0 ± 69.3	I: No—*P *> 0.05 C: No—*P *> 0.05 Inter-group: No—*P *> 0.05
		Isometric leg flexion—peak torque/BW (%)	I: 90.0 ± 18.7 to 90.0 ± 18.7 C: 76.0 ± 20.8 to 90.0 ± 27.7	I: No–*P *> 0.05 C: No–*P *> 0.05 Inter-group: No–*P *> 0.05
		Isokinetic leg extension–peak torque/BW (%)	I: 153.0 ± 29.9 to 160.0 ± 33.7 C: 147.0 ± 58.9 to 173.0 ± 69.3	I: No–*P *> 0.05 C: No–*P *> 0.05 Inter-group: No–*P *> 0.05
		Isokinetic leg flexion–peak torque/BW (%)	I: 70.0 ± 18.7 to 77.0 ± 15.0 C: 61.0 ± 20.8 to 76.0 ± 31.2	I: No–*P *> 0.05 C: No–*P *> 0.05 Inter-group: No–*P *> 0.05
George et al. [31]	I: 3 C: 3	Handgrip (right arm; kg)	I: + 1.8 ± 1.6 C: + 2.4 ± 2.1	I: No—*P *> 0.05 C: No—*P *> 0.05 Inter-group: No—*P *> 0.05
		Quadriceps Strength (kg)	I: + 5.7 ± 1.0 C: + 10.2 ± 8.7	I: No—*P *> 0.05 C: No—*P *> 0.05 Inter-group: No—*P *> 0.05

Mean change refers to changes in a measurement that occurred between the baseline assessment, and the assessment that immediately followed supplement cessation

*I* intervention group, *C* control group, *BW* body weight

^a^Inter-quartile range

### Muscle performance measurements

Five studies assessed changes in muscle performance (6MWT or get up and go test, Table 5) [27, 28, 30–32], however, one of these studies did not quantitatively report intervention or control data [30]. Data on changes in muscle performance were reported in four studies that included *n *= 74 intervention and *n *= 38 control patients. All four studies measured changes in 6MWT distance [27, 28, 31, 32], one of which also reported changes in timed get up and go [31]. One study did not report control data for the 6MWT (*n *= 6) [28] and one was a cohort study (*n *= 13) [32].

**Table 5:** Changes in muscle performance measurements (mean ± SD)

Study	Sample size (*n *=)	Measurement of muscle function	Change in muscle function (SD)	Significant improvement
Aquilani et al. [27]	I: 21 C:17	6MWT (m)	I: 331.0 ± 124.0 to 405.0 ± 130.0 C: 298.0 ± 142.0 to 310.0 ± 155.0	I: Yes—*P *< 0.001 C: No—*P *> 0.05 Inter-group: Yes—*P *= 0.02
Rozentryt et al. [28]	I: 23 C:6	6MWT (m)	I: 366.0 ± 110.0 to 410.0 ± 107.0 C: Not Reported	I: Yes—*P *= 0.020 C: No—*P *> 0.05 Inter-group: Not reported
Wu et al. [30]	I: 14 C: 12	6MWT (m)	I: Numerical values not reported C: Numerical values not reported	I: No—*P *> 0.05 C: No—*P *> 0.05 Inter-group: No—*P *> 0.05
George et al. [31]	I: 3 C: 3	6MWT (m)	I: − 49.0 ± 42.0 C: − 43.0 ± 37.0	I: No—*P *> 0.05 C: No—*P *> 0.05 Inter-group: No—*P *> 0.05
		Get Up and Go (seconds)	I: + 1.8 ± 2.8 C: + 2.8 ± 4.3	I: No—*P *> 0.05 C: No—*P *> 0.05 Inter-group: No—*P *> 0.05
Lombardi et al. [32]	I: 13 C: N/A	6MWT (m)	I: 439.1 ± 64.3 to 474.2 ± 89.0 C: N/A	I: Yes—*P *= 0.006 C: N/A Inter-group: N/A

One study, where only the intervention group was prescribed exercise, did not find an increase in 6MWT distance or timed get up and go among intervention patients (*n *= 3), when compared to control patients (*n *= 3) [31]. Three studies [27, 28, 32] including one cohort study [32], reported a significant improvement in 6MWT distance after the nutritional intervention (*n *= 57). Only one study conducted inter-group statistical comparisons of changes in 6MWT distance. 6MWT distance increased significantly in intervention (331 ± 124 m to 405 ± 130 m), but not control patients (298 ± 142 m to 310 ± 155 m; *P *= 0.02) [27]. The RCT that did not report control data found an increase in 6MWT distance, from 366 ± 110 m to 410 ± 107 m (*P *= 0.020) [28]. In the cohort study, 6MWT distances increased from 439 ± 64 m to 474 ± 89 m (*P *= 0.006) [32]. The RCT that did not quantitatively report outcome data did not find significant between-group differences in 6MWT distance (*P *> 0.05) [30].

### Body mass measurements

The results for body composition measurements are presented in Table 6. Three studies involving *n *= 73 intervention and *n *= 49 control patients reported changes in body mass [27–29]. One study, which prescribed exercise in the intervention and control groups, did not find a significant increase in body mass among intervention patients, when compared to control patients (*P *> 0.05) [29]. One study reported an intra-group body mass increase from 55.9 ± 17.0 kg to 58.2 ± 7.2 kg (*P *< 0.010) [27], however changes in body mass were not significantly greater than those found in the control group (*P *> 0.05). A third study found an increase in intra-group body mass, from 63.9 ± 9.4 kg to 65.5 ± 10.3 kg (*P *< 0.001) following the study intervention. However, no control data were reported and inter-group comparisons were not conducted [28].

**Table 6:** Changes in measurements of body composition (mean ± SD)

Study	Sample size (*n *=)	Measurement of body composition	Change in body composition (SD)	Significant improvement
Aquilani et al. [27]	I: 21 C:17	Arm muscle area (Skin fold measurement-derived; cm^2^)	I: 31.2 ± 9.9 to 34.9 ± 10.0 C: 34.2 ± 5.0 to 37.1 ± 4.0	I: Yes—*P *< 0.020 C: Yes—*P *< 0.020 Inter-group: *P *> 0.05
		Tricep skinfold thickness (mm)	I: 10.4 ± 4.4 to 10.3 ± 3.9 C: 11.9 ± 3.7 to 11.4 ± 3.7	I: No—*P *> 0.05 C: No—*P *> 0.05 Inter-group: No—*P *> 0.05
		Body mass (kg)	I: 55.9 ± 17.0 to 58.2 ± 7.2 C: 60.8 ± 7.0 to 61.2 ± 6.3	I: Yes—*P *< 0.010 C: No—*P *> 0.05 Inter-group: No—*P *> 0.05
		BMI (kg/m^2^)	I: 22.5 ± 2.1 to 23.4 ± 1.9 C: 23.2 ± 1.4 to 23.6 ± 1.5	I: Yes—*P *< 0.010 C: No—*P *> 0.05 Inter-group: No—*P *> 0.05
Rozentryt et al. [28]	I: 23 C:6	Lean body mass (DXA; kg)	I: 45.0 ± 12.5 to 45.5 ± 11.2 C: Not reported	I: Yes—*P *= 0.019 C: No—*P *> 0.05 Inter-group: Not reported
		Fat mass (DXA; kg)	I: 15.6 ± 8.9 to 16.6 ± 8.9 C: Not reported	I: Yes—*P *= 0.003 C: No—*P *> 0.05 Inter-group: Not reported
		Body mass (DXA; kg)	I: 63.9 ± 9.4 to 65.5 ± 10.3 C: Not reported	I: Yes—*P *= 0.003 C: No—*P *> 0.05 Inter-group: Not reported
Pineda-Juarez et al. [29]	I: 29 C:26	Body mass (kg)	I: − 0.6 (− 4.4 to 2.1)^a^ C: -0.5 (− 2.4 to 2.7)^a^	I: No—*P *> 0.05 C: No—*P *> 0.05 Inter-group: No—*P *> 0.05
		BMI (kg/m^2^)	I: − 0.4 (− 2.7 to 2.2)^a^ C: − 0.7 (− 2.1 to 2.3)^a^	I: No—*P *> 0.05 C: No—*P *> 0.05 Inter-group: No *P *> 0.05
		Arm circumference (% change)	I: − 2.0 (− 7.4 to 3.3)^a^ C: − 4.0 (− 7.6 to 0.2)^a^	I: No—*P *> 0.05 C: No—*P *> 0.05 Inter-group: No *P *> 0.05
		Hip circumference (% change)	I: − 3.1 (− 6.6 to − 1.1) C: − 1.5 (− 3.7 to − 1.8)	I: No—*P *> 0.05 C: No—*P *> 0.05 Inter-group: No *P *> 0.05
		Waist circumference (% change)	I: − 0.7 (− 3.2 to − 3.3) C: − 1.7 (− 3.7 to − 1.8)	I: No—*P *> 0.05 C: No—*P *> 0.05 Inter-group: No *P *> 0.05
Wu et al. [30]	I: 14 C: 12	Lean body mass (DXA; kg)	I: 54.4 ± 2.8 to 56.1 ± 2.5 C: 52.4 ± 11.1 to 53.8 ± 12.8	I: Yes—< 0.05 C: No—*P *> 0.05 Inter-group: Yes *P *= 0.040
		Fat mass (DXA; kg)	I: 27.0 ± 7.5 to 26.0 ± 7.5 C: 25.0 ± 6.9 to 26.0 ± 6.9	I: No—*P *> 0.05 C: No—*P *> 0.05 Inter-group: No *P *> 0.05
		BMI (DXA; kg/m^2^)	I: 30.0 ± 1.0 to 30.0 ± 1.0 C: 28.0 ± 2.0 to 29.0 ± 2.0	I: No - *P *> 0.05 C: No - *P *> 0.05 Inter-group: No *P *> 0.05
Lombardi et al. [32]	I: 13 C: N/A	BMI (kg/m^2^)	I: 25.7 ± 3.2 to 25.4 ± 2.8 C: N/A	I: No C: N/A Inter-group: N/A

### Body mass index measurements

Four studies [27, 29, 30] including one cohort study [32] reported data for body mass index [BMI] (*n *= 77 intervention and *n *= 55 control patients). Only one study reported a significant increase in BMI following supplementation (22.5 ± 2.1 kg/m^2^ to 23.4 ± 1.9 kg/m^2^; *P *< 0.010) [27]. However, this change was not significantly different to changes reported in the control group.

### Fat mass measurements

Two studies, including *n *= 37 intervention and *n *= 18 control patients, reported Dual X-ray Absorptiometry (DXA) derived changes in fat mass. One study reported no change in fat mass among intervention group patients, when compared to control group patients (*P *> 0.05) [30]. The second study reported an increase in fat mass, from 15.6 ± 0.7 to 16.6 ± 0.9 (*P *= 0.003) in the intervention group. No control data were reported and inter-group comparisons were not conducted [28].

### Lean mass measurements

Two studies, including *n *= 37 intervention and *n *= 18 control patients reported DXA derived changes in lean body mass [30]. Wu and Colleagues [30] reported that lean body mass significantly increased when compared to controls (*P *= 0.04). Rozentryt and colleagues [28] reported that lean body mass increased among intervention patients (*P *= 0.019), but did not report control data.

One study (intervention arm *n *= 21; control arm *n *= 17) estimated lean body mass using tricep skinfold thickness measurements and arm muscle area (cm^2^) [27]. Tricep skinfold thickness measurements did not change in either study groups (*P* value not reported). Arm muscle area increased in the intervention and control groups (*P *= 0.020). One study (intervention arm *n *= 29; control arm *n *= 26; exercise prescribed to both groups) reported arm circumference as an estimate of lean body mass [29]. There was no improvement in arm circumference among intervention group patients, when compared to control group patients (*P *> 0.05).

### Aerobic fitness measurements

Five studies including *n *= 100 intervention and *n *= 61 control patients assessed changes in aerobic fitness (Table 7) [27–30, 32]. One study did not report control data [28], one was a cohort study [32], and one prescribed exercise to the intervention and control groups [29]. Three studies did not find any changes in either estimated (*P *> 0.05) [29], or directly determined peak oxygen uptake (V̇O_2peak_) following protein and/or EAA supplementation (*P *= 0.320 [28] and *P *= 0.260 [30]). One study reported a significant increase in V̇O_2peak_ (*P *< 0.050) and peak power (*P *< 0.010) output among intervention patients, when compared to control patients [27]. The cohort study reported significant improvements in V̇O_2peak_ (*P *= 0.008) and the ventilatory anaerobic threshold (*P *= 0.002), but not peak power output (*P *= 0.380) or ventilatory efficiency (V̇E/V̇CO_2_ slope; *P *= 0.754) [32].

**Table 7:** Changes in measurements of aerobic fitness (mean ± SD)

Study	Sample size (*n *=)	Measurement of aerobic fitness	Change in aerobic fitness (SD)	Significant improvement
Aquilani et al [27]	I: 21 C:17	V̇O_2peak_ (ml/kg/min)	I: 13.5 ± 1.7 to 14.9 ± 1.9 C: 12.9 ± 2.7 to 13.0 ± 3.5	I: Yes—*P *< 0.050 C: No—*P *> 0.050 Inter-group: Yes—*P *< 0.050
		Peak power output (w)	I: 80.0 ± 28.0 to 95.0 ± 25.0 C: 85.0 ± 24.0 to 88.0 ± 22.0	I: Yes—*P *< 0.020 C: No—*P *> 0.050 Inter-group: Yes—*P *< 0.010
Rozentryt et al. [28]	I: 23 C:6	V̇O_2peak_ (ml/kg/min)	I: 14.5 ± 2.9 to 14.9 ± 3.1 C: Not reported	I: No—*P *= 0.320 C: No—*P *> 0.050 Inter-group: Not reported
Pineda-Juarez et al. [29]	I: 29 C:26	Estimated V̇O_2peak_ (% change)	I: + 16.6 (0.2 to 38.5)^a^ C: 50.1 (− 11.2 to 94.0)^a^	I: No—*P *> 0.05 C: No—*P *> 0.05 Inter-group: No—*P *> 0.05
Wu et al. [30]	I: 14 C: 12	V̇O_2peak_ (ml/kg/min)	I: + 7.9 ± 17.6 C: + 0.1 ± 2.6%	I: No—No—*P *> 0.05 C: No—*P *> 0.05 Inter-group: No—*P *= 0.260
Lombardi et al. [32]	I: 13 C: N/A	V̇O_2peak_ (ml/kg/min)	I:14.8 ± 3.9 to 16.8 ± 5.1 C: N/A	I: Yes—*P *= 0.008 C: N/A Inter-group: N/A
		VAT (ml/kg/min)	I: 9.0 ± 3.8 to 12.4 ± 3.9 C:N/A	I: Yes—*P *= 0.002 C: N/A Inter-group: N/A
		V̇E/VCO_2_ slope	I: 37.1 ± 6.9 to 37.4 ± 7.7 C: N/A	I: No - *P *= 0.754 C: N/A Inter-group: N/A
		Peak power output (w)	I: 100.9 ± 32.4 to 104.8 ± 28.4	I: No—*P *= 0.380 C: N/A Inter-group: N/A

### Health related quality of life measurements

Four studies reported changes in patient HRQoL [28, 30–32]. Three, including one cohort study [32] assessed HRQoL using the Minnesota living with heart failure questionnaire (MLHFQ) [30, 32, 36]. Control data were not reported for either RCT. Two (of three) studies [28, 32] reported an improvement in MLHFQ scores. Rozentryt and colleagues [28] reported a change from 47 ± 23 to 37 ± 27 (*P *< 0.001; control data not reported), whilst Wu and colleagues [30] reported an improvement from 36 ± 82 to 24 ± 57 in the intervention, but not control group (*P *= 0.020; control data not reported). The same study also assessed changes in HRQoL using the Kansas City Cardiomyopathy Questionnaire (KCCQ) [37]. They identified an overall improvement (73 ± 71 to 83 ± 45; *P *= 0.040), improvements in social limitation (72 ± 90 to 86 ± 56; *P *= 0.006), and HRQoL (62 ± 101 to 75 ± 60; *P *= 0.004). Lombardi and colleagues [32] did not find an improvement in MLHFQ score (21 ± 14 to 25 ± 13; *P *= 0.321).

One study [31], which combined exercise with supplementation in the intervention group only, reported changes in HRQoL using the Short Form Health Survey (SF-36) questionnaire [38]. Only scores for individual questionnaire items were reported. There were no significant changes in SF-36 scores for patients in the intervention (*n *= 3) or control groups (*n *= 3). Cumulative SF-36 scores and SEM values were not reported.

### Safety

Four studies reported serious adverse event and adverse event data (Table 2) [27–30]. There were 21 serious adverse events. Eight of the serious adverse events were deaths [27–29] (4.8% of study population) [27, 28]. Four of the deaths were among intervention patients, and four among controls. One study involving exercise training for intervention and control patients reported two deaths in each study arm, however the cause of death was not reported (total deaths *n *= 4) [29].The absolute number of serious adverse events (*n *= 13) was higher among intervention patients compared to controls (*n *= 8). However, due to a higher sample in the intervention compared to control group, the serious adverse event rate was proportionally similar among intervention and control patients, with one serious adverse event for every eight patients recruited. There were a total of 28 adverse events. Adverse events affected one in five patients assigned to intervention groups (total; *n* = 19), and one in seven patients assigned to control groups (total; *n *= 9). One study comprehensively reported adverse and serious adverse events [28]. The serious adverse events occurring in this study accounted for 52% of all serious adverse events (*n *= 14), and 72% of adverse events (*n *= 21) reported in our systematic review. Renal function was reported using estimated glomerular filtration rate (two studies) [29, 32], serum creatinine levels (four studies) [28–30, 32] and serum albumin levels (three studies) [28–30]. No changes in renal function were reported.

### Study attrition

All six studies reported study attrition (Online Resource 5) [27–32]. Study attrition was similar among intervention and control arms. Attrition in the intervention arms ranged from 0% to 50%, and attrition in the control arms ranged from 0% to 40%.

### Study adherence

Only one study reported supplementation adherence (Online Resource 5) [27]. Adherence was defined by the total number of empty supplement packets returned by the participant at the end of the intervention. Adherence was further evidenced by blood sample analyses, leucine (EAA) concentrations within the intervention group were 279% higher than reported at baseline (*P *< 0.001). Based on this, the authors concluded that all participants adhered to the intervention.

## Discussion

### Overview

The primary aim of this systematic review was to assess the effects of dietary protein and/or EAA supplementation on skeletal muscle strength, and performance in patients with CHF. The number of studies (*n *= 6) and total patient population (*n *= 167) were small, and the risk of bias was high in all RCTs. Methods of assessing our outcomes of interest were also heterogeneous, and data were often incompletely reported. For example, in most cases, only two studies reported the same outcome measure, and pooled sample sizes were small. In cases where three or more studies appeared to report the same outcome measure, complete intervention and control outcome data were only reported by two studies. Furthermore, data from the cohort study could not be pooled with data from the RCTs. The heterogeneous nature of these studies meant that meta-analysis was inappropriate [33]. Qualitative data synthesis suggested that dietary supplementation with protein and/or EAA may not improve strength in patients with CHF, [29–31] but may increase muscle performance (6MWT distance) [27, 28, 32]. The secondary findings showed that protein/EAA supplementation may improve lean body mass [28, 30], health-related quality of life and appears to be safe. There is limited evidence for the intervention improving aerobic fitness.

### Strength measurements

Recent changes to sarcopenia guidelines advocate primarily identifying low muscle strength [10] instead of lean or fat-free body mass [39] for diagnosis. Developing an intervention that can increase strength and avoid or defer the development of sarcopenia in patients with CHF is required. We identified three studies that measured changes in muscle strength, none of which reported a significant improvement following protein and/or EAA supplementation despite two of the studies employing a combined resistance exercise and protein/EAA intervention. Similar findings have previously been reported in non-frail elderly individuals [40], however evidence appears to support the beneficial role of protein and/or EAA supplementation in increasing strength in healthy adults [16] and undernourished people with a long-term condition [17].

A review of 49 studies by Morton and colleagues [16] reported a mean 1 repetition maximum (1RM) strength increase of 27.0 kg (95% CI 22.0–32.0 kg) following combined resistance exercise training and protein supplementation in healthy adults. Dietary protein supplementation resulted in a further modest 1RM increase of 2.5 kg (95% CI 0.6–4.3 kg), indicating that protein supplementation plays a smaller role in improving strength, in comparison to resistance exercise training. Similarly, a systematic review by Cheng and colleagues [17] found that dietary protein and/or amino acid supplementation elicited modest improvements in strength in people with a long-term condition (standardised mean difference [SMD]: 0.27 kg; 95% CI 0.1–0.4 kg; *P *< 0.01) [17]. These findings appeared more pronounced in individuals who were undernourished. Interestingly, only one study identified by Cheng and colleagues [27] specifically recruited patients with CHF. Our findings confirm that there is a paucity of data in this population that has a high incidence of sarcopenia.

Similar to our review, the review by Cheng and colleagues [17] extracted data from a study by Aquilani and colleagues [27]. Details of this intervention are reported in Table 2. Aquilani and colleagues measured aerobic fitness on a cycle ergometer. However, this was interpreted to be a measure of strength by Cheng and colleagues. Peak aerobic fitness is determined by oxygen transport to the muscle and muscle oxygen extraction [41] and may not reflect changes in muscular strength. Despite this important discrepancy, the findings of systematic reviews on protein and/or EAA supplementation in older adults with [17] or without [40] long-term conditions lend support to our observation that protein and/or EAA supplementation either does not improve or has a small effect on improving muscular strength [17, 40]. This may be because muscular strength is dependent on neurological activation of the muscle, as well as increases in lean muscle mass [42]. Protein and/or EAA supplementation alone may not stimulate improvements in motor unit recruitment required for strength development. Mechanical stimuli, such as that provided by resistance exercise training, may be required to optimise the potential benefits that supplementation may provide.

We identified two studies that measured changes in strength following protein and/or EAA supplementation and exercise training. However, the study design limited the ability to adequately evaluate whether this combined approach is effective in improving strength. George et al. [31] assessed the feasibility of protein supplementation and exercise training in patients with CHF [31]. No significant improvements in strength were reported, however, the study was not powered to detect statistically significant changes in physiological outcomes (*n *= 3 per study group) [31]. Pineda-Juarez and colleagues [29] also reported no improvement in strength among patients who were randomised to EAA supplementation or control groups. However, this study was limited by patients having an average energy intake of ~ 1423 kcal per day; a substantially lower dietary energy intake than is currently recommended for adult males and females (~ 2000 to ~ 2500 kcal) [43]. Low dietary energy intake combined with increased energy expenditure with resistance exercise training may have exacerbated the catabolic nature of CHF [44]. Furthermore, Pineda-Juarez et al. [29] removed 10 g of protein from intervention patients’ diets, before providing the twice daily 5 g non-EAA and EAA mixture. Each 5 g supplement, however, only contained 3.75 g of non-EAA and EAA’s. Compared to baseline, patients in the intervention group therefore consumed 2.5 fewer grams of protein per day than the control group, for the duration of the study [29]. Collectively, these factors may limit any expected improvements in strength, among patients assigned to the intervention group. Protein/EAA supplementation does not appear to improve strength of patients with CHF.

### Body mass measurements

Three of five studies that measured body mass, reported an increase in at least one surrogate marker of lean muscle mass (arm muscle area, arm circumference and, BMI) [27, 28, 30]. Two studies also reported an increase in DXA-derived measurements of lean body mass [28, 30], the reference standard for measuring body mass in sarcopenia [45]. Similar to data reported in healthy older people [16] and people living with a long-term condition [17], protein and/or EAA supplementation in patients with CHF led to an increase in lean body mass (DXA: 0.5–1.7 kg) [28, 30]. Protein/EAA supplementation appears to be beneficial in improving lean mass.

Despite increases in lean mass, Wu and colleagues [30] did not report any changes in fat mass, BMI or lipid profiles, suggesting that cardiometabolic health was maintained throughout the supplementation period. This study increased dietary energy intake by 90 kcal per day. However, Rozentryt and colleagues [28] reported that a 300 kcal twice daily multi-macronutrient supplementation (20 g of protein, 72 g carbohydrate and 26 g of fat) led to an increase in total body mass and fat mass [28]. Given that 600 kcal/day constitutes approximately 35–45% of the daily energy expenditure of CHF patients [46], it is unsurprising that such an increase in dietary intake for 3 months led to increases in lean mass and fat mass.

Previous evidence suggests that mortality risk [47] and cardiac stress markers (NT-proBNP) [48] are lower in CHF patients who are overweight (BMI of up to 29 kg/m^2^). Therefore, one might assume that the increases in body mass reported by Rozentryt and colleagues [25] are beneficial. However, high fat mass could be detrimental in CHF due to the observed higher level of inflammation (high-sensitivity C-reactive protein) and lower 6MWT distances [48]. High levels of inflammation may increase the risk of developing sarcopenia, and is associated with the progression of CHF [10, 49, 50]. Furthermore, greater six-minute walk test distances confer superior survival outcomes [51] and better HRQoL in patients with CHF [52]. Increasing body mass, without increasing fat mass may help to preserve 6MWT distance and consequently, HRQoL in people with CHF. Improved lean and total body masses without increased fat mass may be achieved with small increases in caloric intake (90 kcal) by predominantly supplementing with pro-anabolic EAA and non-EAAs [30].

### Muscle performance

Three of the four studies that measured 6MWT distance reported a significant improvement among intervention patients, but not controls. The only study that did not report a significant improvement was a feasibility study with low statistical power (*n *= 3 per group) [31]. Change in 6MWT distance exceeded the widely accepted minimally important improvement for people with CHF (43 m) [53] in two studies [27, 28]. These improvements are consistent with the effects of protein and/or EAA supplementation in people with a long-term condition [17] and similar to improvements reported after exercise-based cardiac rehabilitation in patients with CHF (46 m; *P *< 0.001) [54].

It is noteworthy that the gold standard measurement of aerobic fitness, V̇O_2peak_, increased in only two out of five studies [27, 32]. This may suggest that protein and/or EAA supplementation is more likely to improve sub-maximal muscle performance, rather than maximal aerobic fitness. Protein and EAA supplementation appears to improve muscle performance assessed by walking distance.

### Health-related quality of life measurements

Changes in HRQoL were typically reported using the MLHFQ [28, 30] and the KCCQ [30]. The MLHFQ and KCCQ require patients to provide responses to questions relating to the impact that CHF has on their physical, emotional and socio-economic function. They are widely adopted in clinical trials involving patients with CHF and are likely to be sensitive to changes in frailty. However, to our knowledge, their sensitivity to changes in strength, muscle mass or performance has not been investigated. Therefore, it is possible that changes in HRQoL may not have been adequately captured. It is noteworthy that none of the studies in our systematic review used qualitative methods to explore changes in HRQoL. Qualitative research methods can provide important context to HRQoL data obtained from questionnaires and can help researchers to interpret quantitative findings [55]. Qualitative research methods, such as patient interviews, could help to further our understanding of the impact the protein and/or EAA supplementation has on HRQoL. Based on the available evidence, protein and/or EAA supplementation may lead to an improvement in HRQoL.

### Attrition

In a RCT, high levels of patient attrition can increase the likelihood of a biased outcome and misleading findings [56]. Five out of six studies in our systematic review had an attrition rate  < 20% [27–30, 32]. Attrition rates between 5% and 20% are likely to lead to a modest risk of outcome bias in RCTs [57]. The observed low to modest risk of bias suggests that adequately powered RCTs, investigating protein and/or EAA supplementation, may be feasible.

### Adherence

Recording adherence to nutritional supplementation in home-based interventions can be difficult. Only one study reported intervention adherence in our systematic review [27]. Adherence was estimated by counting the number of empty supplement packets those were returned to the research team and the increases in plasma EAA concentractions. It is promising that Aquilani and colleagues [27] found that 100% of patients adhered to their intervention. However, detailed information on adherence to protein and/or EAA supplementation in patients with CHF is required to determine whether patients are likely to adhere to protein and/or EAA supplementation in clinical practice.

### Safety

Diets that are high in protein have been associated with a decline in renal function over a 41-month period, in patients with heart disease [24]. Therefore, the benefits of treating sarcopenia in patients with CHF using protein and/or EAA supplementation need to be evaluated against the risk of a decline in renal function. The data available in our systematic review suggest that interventions using protein and/or EAA supplementation that last up to 3 months [28, 30, 32] may not have a negative effect on renal function. However, this finding should be interpreted with caution, because adverse event reporting was often incomplete. Available adverse event data indicate that a proportionately similar number of adverse events were experienced by patients in intervention and control arms, and no significant safety concerns have been identified in our systematic review. However, it is essential that future clinical trials in this field of research report adverse events in a transparent way [22].

### Limitations

The paucity of studies that supplemented CHF patients with protein and/or EAA with the aim of improving muscular strength or performance limited the strength of our findings. Furthermore, the risk of bias was high, outcome measures were heterogeneous and the quality of data reporting was varied, and often incomplete. This precluded meta-analysis, and therefore estimation of the intervention effect size. The conclusions that can be drawn from our findings are therefore limited. Qualitative interpretation of the studies included in our review also indicated that a number of studies had design limitations. These may have influenced the outcomes of the studies and consequently our systematic review. One important design limitation was that only two studies prescribed and recorded exercise participation during the study. Exercise is a potent stimulator of muscle anabolism and strength, and patients with CHF are advised to participate in structured exercise training, however, participation is highly variable. Not recording or controlling patient participation in structured exercise training may attenuate the overall effect signal for the protein/EAA intervention.

### Future studies

It is clear from the small number of studies included in this review, and their recent publication dates, that this field of research is in its infancy. Future research should take into consideration the high risk of bias and low quality of the study methods observed in the studies included in this review. To improve the quality of future research, transparent reporting of adverse events, reporting changes in renal function, adherence to supplementation regimens, and qualitatively exploring patient experiences of participating in a protein and/or EAA supplementation regimen should be considered as priorities for future studies. Further qualitative research is needed to increase our understanding of how protein and/or EAA supplementation may improve HRQoL in patients with CHF. This may be achieved, in part, by validation of the MLHFQ and KCCQ questionnaires against measurements of sarcopenia. Alternatively, validating sarcopenia specific questionnaires such as the recently developed SarQoL [58] in patients with CHF may provide additional information on changes in HRQoL following protein and/or EAA supplementation. Researchers should also consider whether using protein and/or EAA supplementation results in a significant increase in daily calorie intake, and what effect this may have on markers of cardiometabolic health, inflammation and muscle performance. Finally, further research is needed to explore whether exercise training may augment any benefits associated with protein and/or EAA supplementation for the treatment of sarcopenia in patients with CHF.

## Conclusions

Protein and/or EAA supplementation appears to be safe, may increase lean body mass and 6MWT distance, but not strength in patients with CHF. Trials reporting changes in strength had substantial limitations and these findings should be interpreted with caution. Combining strength training with protein supplementation optimises muscle strength and performance adaptations in healthy older participants. Research investigating the benefits of protein and/or EAA supplementation combined with resistance exercise training in patients with CHF is needed.

## Electronic supplementary material

Below is the link to the electronic supplementary material.

Supplementary material 1 (DOCX 34 kb)
