# Supplementary material for: The effect of protein and essential amino acid supplementation on muscle strength and performance in patients with chronic heart failure: a systematic review
Source: Eur J Nutr. 2019 Oct 28;59(5):1785–801. doi: 10.1007/s00394-019-02108-z (PMC7351803; doi:10.1007/s00394-019-02108-z)
Supplement: Supplementary file 1 — Supplementary material 1 (DOCX 34 kb) [file 394_2019_2108_MOESM1_ESM.docx]

European Journal of Nutrition

The effect of protein and amino acid supplementation on muscle mass and function in patients with chronic heart failure – A systematic review

^*^Simon Nichols^1^, Gordon McGregor^2,3^, Abdallah Al-Mohammad^4^, Ali N Ali^5^, Garry Tew^6^, Alasdair F O’Doherty^6^

*Corresponding Author

Dr Simon Nichols

^1^Centre for Sports and Exercise Science,

Sheffield Hallam University,

Collegiate Campus,

Sheffield,

United Kingdom,

S10 2BP

E-mail: [s.j.nichols@shu.ac.uk](mailto:s.j.nichols@shu.ac.uk)

Tel: 01142 254327

Fax: None

Dr Gordon McGregor

^2^Department of Cardiopulmonary Rehabilitation,

Centre for Exercise & Health,

University Hospitals Coventry & Warwickshire NHS Trust,

Coventry,

United Kingdom

^3^School of Health & Life Sciences,

Coventry University,

Coventry,

United Kingdom

Dr Abdallah Al-Mohammad

^4^Sheffield Teaching Hospitals NHS Foundation Trust

Herries Rd,

Sheffield

United Kingdom,

Dr Ali N Ali

^5^Sheffield National Institute for Health Research Biomedical Research Centre

Glossop Road,

Sheffield,

United Kingdom

Assoc. Professor Garry Tew and Dr Alasdair F O’Doherty

^6^Department of Sport, Exercise and Rehabilitation,

Northumbria University,

Newcastle-Upon-Tyne,

United Kingdom,

**Online Resource 1** – PRISMA Checklist

| **Section/topic** | **#** | **Checklist item** | **Reported on page #** |
| --- | --- | --- | --- |
| **TITLE** | | |  |
| Title | 1 | Identify the report as a systematic review, meta-analysis, or both. | 2 |
| **ABSTRACT** | | |  |
| Structured summary | 2 | Provide a structured summary including, as applicable: background; objectives; data sources; study eligibility criteria, participants, and interventions; study appraisal and synthesis methods; results; limitations; conclusions and implications of key findings; systematic review registration number. | 5 |
| **INTRODUCTION** | | |  |
| Rationale | 3 | Describe the rationale for the review in the context of what is already known. | 6-7 |
| Objectives | 4 | Provide an explicit statement of questions being addressed with reference to participants, interventions, comparisons, outcomes, and study design (PICOS). | 7-8 |
| **METHODS** | | |  |
| Protocol and registration | 5 | Indicate if a review protocol exists, if and where it can be accessed (e.g., Web address), and, if available, provide registration information including registration number. | 7 |
| Eligibility criteria | 6 | Specify study characteristics (e.g., PICOS, length of follow-up) and report characteristics (e.g., years considered, language, publication status) used as criteria for eligibility, giving rationale. | 7-8 |
| Information sources | 7 | Describe all information sources (e.g., databases with dates of coverage, contact with study authors to identify additional studies) in the search and date last searched. | 8 |
| Search | 8 | Present full electronic search strategy for at least one database, including any limits used, such that it could be repeated. | 7 and online resource 2 |
| Study selection | 9 | State the process for selecting studies (i.e., screening, eligibility, included in systematic review, and, if applicable, included in the meta-analysis). | 7-9 |
| Data collection process | 10 | Describe method of data extraction from reports (e.g., piloted forms, independently, in duplicate) and any processes for obtaining and confirming data from investigators. | 9-10 |
| Data items | 11 | List and define all variables for which data were sought (e.g., PICOS, funding sources) and any assumptions and simplifications made. | 9-10 |
| Risk of bias in individual studies | 12 | Describe methods used for assessing risk of bias of individual studies (including specification of whether this was done at the study or outcome level), and how this information is to be used in any data synthesis. | 10-11 |
| Summary measures | 13 | State the principal summary measures (e.g., risk ratio, difference in means). | 10 |
| Synthesis of results | 14 | Describe the methods of handling data and combining results of studies, if done, including measures of consistency (e.g., I^2^) for each meta-analysis. | 10 |

Page 1 of 2

| **Section/topic** | **#** | **Checklist item** | **Reported on page #** |
| --- | --- | --- | --- |
| Risk of bias across studies | 15 | Specify any assessment of risk of bias that may affect the cumulative evidence (e.g., publication bias, selective reporting within studies). | 10 |
| Additional analyses | 16 | Describe methods of additional analyses (e.g., sensitivity or subgroup analyses, meta-regression), if done, indicating which were pre-specified. | 9-10 |
| **RESULTS** | | |  |
| Study selection | 17 | Give numbers of studies screened, assessed for eligibility, and included in the review, with reasons for exclusions at each stage, ideally with a flow diagram. | 11 |
| Study characteristics | 18 | For each study, present characteristics for which data were extracted (e.g., study size, PICOS, follow-up period) and provide the citations. | 11-12 |
| Risk of bias within studies | 19 | Present data on risk of bias of each study and, if available, any outcome level assessment (see item 12). | 12 |
| Results of individual studies | 20 | For all outcomes considered (benefits or harms), present, for each study: (a) simple summary data for each intervention group (b) effect estimates and confidence intervals, ideally with a forest plot. | 12-19 |
| Synthesis of results | 21 | Present results of each meta-analysis done, including confidence intervals and measures of consistency. | n/a |
| Risk of bias across studies | 22 | Present results of any assessment of risk of bias across studies (see Item 15). | 10 |
| Additional analysis | 23 | Give results of additional analyses, if done (e.g., sensitivity or subgroup analyses, meta-regression [see Item 16]). | n/a |
| **DISCUSSION** | | |  |
| Summary of evidence | 24 | Summarize the main findings including the strength of evidence for each main outcome; consider their relevance to key groups (e.g., healthcare providers, users, and policy makers). | 19 |
| Limitations | 25 | Discuss limitations at study and outcome level (e.g., risk of bias), and at review-level (e.g., incomplete retrieval of identified research, reporting bias). | 26 |
| Conclusions | 26 | Provide a general interpretation of the results in the context of other evidence, and implications for future research. | 19-25 |
| **FUNDING** | | |  |
| Funding | 27 | Describe sources of funding for the systematic review and other support (e.g., supply of data); role of funders for the systematic review. | 4 |

*From:*  Moher D, Liberati A, Tetzlaff J, Altman DG, The PRISMA Group (2009). Preferred Reporting Items for Systematic Reviews and Meta-Analyses: The PRISMA Statement. PLoS Med 6(6): e1000097. doi:10.1371/journal.pmed1000097

For more information, visit: **www.prisma-statement.org**.

Page 2 of 2

**Online Resource 2 - Search Strategy**

**Embase:**

1. "heart failure".ti,ab

2. HEART FAILURE/

3."left ventricular failure".ti,ab

4. "cardiac failure".ti,ab

5. 1 OR 2 OR 3 OR 4

6. "cachexia".ti,ab

7. CACHEXIA/

8. SARCOPENIA/

9. "sarcopenia".ti,ab

10. "skeletal muscle".ti,ab

11. "lean mass".ti,ab

12. "muscle mass".ti,ab

13. 6 OR 7 OR 8 OR 9 Or 10 OR 11 OR 12

14. "Amino acids".ti,ab

15. "AMINO ACIDS"/

16. ("Branch* Chain Amino Acid").ti,ab

17. "BRANCHED-CHAIN AMINO ACIDS"/ OR "AMINO ACID"/

18. ("Protein").ti,ab

19. 14 OR 15 OR 16 OR 17 OR 18

20. 5 AND 13 AND 19

**PubMed:**

1. "heart failure".ti,ab

2. "cardiac failure".ti,ab

3."left ventricular failure".ti,ab

4. 1 OR 2 OR 3

5. "cachexia".ti,ab

6. "sarcopenia".ti,ab

7. "skeletal muscle".ti,ab

8. "lean mass".ti,ab

9. "muscle mass".ti,ab

10. 5 OR 6 OR 7 OR 8 OR 19

11. "Amino acids".ti,ab

12. ("Branch* Chain Amino Acid").ti,ab

13. ("Protein").ti,ab

14. 11 OR 12 OR 13

15. 4 AND 10 AND 14

**Medline**

1. "heart failure".ti,ab

2. HEART FAILURE/

3."left ventricular failure".ti,ab

4. "cardiac failure".ti,ab

5. 1 OR 2 OR 3 OR 4

6. "cachexia".ti,ab

7. CACHEXIA/

8. SARCOPENIA/

9. "sarcopenia".ti,ab

10. "skeletal muscle".ti,ab

11. "lean mass".ti,ab

12. "muscle mass".ti,ab

13. 6 OR 7 OR 8 OR 9 Or 10 OR 11 OR 12

14. "Amino acids".ti,ab

15. "AMINO ACIDS"/

16. ("Branch* Chain Amino Acid").ti,ab

17. "AMINO ACIDS, BRANCHED-CHAIN"/

18. ("Protein").ti,ab

19. 14 OR 15 OR 16 OR 17 OR 18

**20. 5 AND 13 AND 19**

**Online Resource 3** - Mixed Methods Appraisal for Lombardi et al ^26^

| **Questions** | **Responses** | | | |
| --- | --- | --- | --- | --- |
|  | **Y** | **N** | **?** | **Comment** |
| First author and year | Lombardi et al 2014 | | | |
| Are there clear qualitative and quantitative research questions (or objectives*), or a clear mixed methods question (or objective*)? | Y |  |  | Effect of intervention (11 different AA’s supplementation) on functional capacity in CHF (VO2 max and 6MWT). |
| Do the collected data allow address the research question (objective)? E.g., consider whether the follow-up period is long enough for the  outcome to occur (for longitudinal studies or study components). | Y |  |  | Although only 13 patients enrolled, open labelled, therefore prone to bias. |
| 1.1 |  |  |  | N/A |
| 1.2 |  |  |  | N/A |
| 1.3 |  |  |  | N/A |
| 1.4 |  |  |  | N/A |
| 2.1 |  |  |  | N/A |
| 2.2 |  |  |  | N/A |
| 2.3 |  |  |  | N/A |
| 2.4 |  |  |  | N/A |
| 3.1 Are participants (organizations) recruited in a way that minimizes selection bias? |  | N |  | Although reports consecutive patients enrolled, there is no mention of the time period for recruitment and thus no way to evaluate if the number recruited really does represent all consecutive patients. The text suggests this is a convenience sample. |
| 3.2 re measurements appropriate (clear origin, or validity known, or standard instrument; and absence of contamination between groups  when appropriate) regarding the exposure/intervention and outcomes? | Y |  |  | Measurements are clear, quantitative and validated measures in patients with heart failure (VO2 max and 6MWT). |
| 3.3 In the groups being compared (exposed vs. non-exposed; with intervention vs. without; cases vs. controls), are the participants  comparable, or do researchers take into account (control for) the difference between these groups? |  |  | NA | Single arm study. Patients themselves act as controls in pre and post study. |
| 3.4 Are there complete outcome data (80% or above), and, when applicable, an acceptable response rate (60% or above), or an acceptable  follow-up rate for cohort studies (depending on the duration of follow-up)? | Y |  |  | 100% complete outcome data at pre-specified time-point. |
| 4.1 |  |  |  | N/A |
| 4.2 |  |  |  | N/A |
| 4.3 |  |  |  | N/A |
| 4.4 |  |  |  | N/A |
| 5.1 |  |  |  | N/A |
| 5.2 |  |  |  | N/A |
| 5.3 |  |  |  | N/A |

**Online Resource 4** –Excluded studies

| Study | Year | intervention | Intervention Duration | Reason for Exclusion |
| --- | --- | --- | --- | --- |
| Machhi et al | 2010 | Amino Acid Supplementation only | 3 Months | Did not measure strength or muscle performance |
| Aquilani et al | 2008 | Amino Acid Supplementation only or Standard Care +Placebo | 30 Days | Did not measure strength or muscle performance |
| Scognamiglio et al | 2008 | Amino Acid Supplementation only or Standard Care +Placebo | 6 Months | Did not measure strength or muscle performance |
| Mancini et al | 1992 | Amino Acid Supplementation only or Standard Care +Placebo | 6 Months | Did not measure strength or muscle performance |
| Azuma et al | 1985 | Amino Acid Supplementation only or Standard Care +Placebo | 4 weeks | Did not measure strength or muscle performance |
| Anand et al | 1998 | Amino Acid Supplementation only or Standard Care +Placebo | 30 days | Did not measure strength or muscle performance |
| Caponnetto et al | 1994 | Amino Acid Supplementation only or Standard Care +Placebo | 6 Months | Did not measure strength or muscle performance |

**Online Resource 5** - Intervention Attrition and Adherence

| **Study** | **Participants Recruited (*n=*)** | **Attrition** | **Compliance** |
| --- | --- | --- | --- |
| Aquilani et al. (22) | I: 22  C:22 | I: 1 (5%)  C: 4 (18%) | I: 100%  C: 100% |
| Rozentryt et al. (23) | I: 23  C: 6 | I: 1 (4%)  C: 0 (0%) | Not Report |
| Pineda-Juares et al. (24) | I:34  C:32 | I: 3 (9%)  C: 4 (13%)" | Not Report |
| Wu et al. (25) | I:17  C:14 | I: 3 (18%)  C: 2 (14%)" | Not Report |
| George et al. (26) | I:6  C:5 | I: 3 (50%)  C:2 (40%)" | Not Report |
| Lombardi et al. (27) | I:6  C:N/A | I: 0 (0%)  C: N/A | Not Report |

I = Intervention Group; C = Control Group;
